# Amoxicillin and Ceftriaxone as Treatment Alternatives to Penicillin for Maternal Syphilis

**DOI:** 10.3201/eid2305.161936

**Published:** 2017-05

**Authors:** Yuichi Katanami, Takehiro Hashimoto, Saho Takaya, Kei Yamamoto, Satoshi Kutsuna, Nozomi Takeshita, Kayoko Hayakawa, Shuzo Kanagawa, Norio Ohmagari

**Affiliations:** National Center for Global Health and Medicine, Tokyo, Japan

**Keywords:** syphilis, maternal syphilis, congenital syphilis, Treponema pallidum, bacteria, penicillin, benzathine penicillin G, amoxicillin, ceftriaxone, probenecid, pregnancy, sexually transmitted infections, newborns, Japan

## Abstract

There is no proven alternative to penicillin for treatment of maternal syphilis. We report 2 case-patients with maternal syphilis who were successfully treated without penicillin. We used amoxicillin and probenecid for the first case-patient and amoxicillin, probenecid, and ceftriaxone for the second case-patient.

Syphilis is caused by the bacterium *Treponema pallidum*. Untreated maternal syphilis can lead to serious complications, including congenital syphilis, stillbirth, and neonatal death (*1*). In 2012, the estimated numbers of worldwide cases of maternal and congenital syphilis were 930,000 and 350,000, respectively (*1*). Recently, the United States reported increasing rates of congenital syphilis (*2*).

Penicillin G is recommended for treatment of maternal syphilis (*3*). Benzathine penicillin G is used in many countries but is unavailable in others, such as Japan. As a result, guidelines in Japan of treatment sexually transmitted diseases recommend benzylpenicillin benzathine hydrate (1.2 million units/d) and oral amoxicillin or ampicillin (1.5 g/d) as alternatives (*4*). However, there is little evidence to support use of these regimens. We report 2 case-patients with maternal syphilis who were successfully treated without penicillin.

## The Study

Case-patient 1 was a 20-year-old woman who came to the hospital at the National Center for Global Health and Medicine (Tokyo, Japan) because of positive results for treponemal and nontreponemal tests in December 2014. Eight months earlier, she was given a diagnosis of trichomonal vaginitis, which resolved after a course of metronidazole. Three months before coming to the hospital, she was examined in a clinic because of a rash on her abdomen and back, for which she was given topical treatment, which resulted in resolution of the rash within a few weeks. A rapid plasma reagin (RPR) and *T. pallidum* hemagglutination assay (TPHA) were not performed at that time.

Two months before coming to the hospital, she missed her menstrual period and showed a positive result for a home pregnancy test. Serologic tests for treponemal and nontreponemal antibodies were performed at a nearby clinic, and she was referred to our hospital 13 weeks into her pregnancy. Her RPR titer was 1:16, and TPHA showed positive results. The patient was given a diagnosis of early latent syphilis and prescribed a 14-day course of amoxicillin (6 g/day) and probenecid (1 g/d). The RPR titer decreased to 1:8 and 1:4 at 3 and 6 months after treatment, respectively. RPR was the last test performed before delivery.

She gave birth to a boy at 41 weeks gestation. The baby did not have any signs or symptoms of congenital syphilis, and his serum RPR and TPHA titers were 1:1 and 1:640, respectively (Table). The baby was not treated for congenital syphilis and has not shown any signs of congenital syphilis infection. At 15 months of age, results for RPR and TPHA were negative for a serum sample from the infant.

**Table:** Characteristics of 2 newborns of women with maternal syphilis, Japan*

Characteristic	Newborn for case-patient 1	Newborn for case-patient 2
Leukocytes, ×1,000 cells/μL	13.78	22.88
Neutrophils, %	76.0	68.0
Lymphocytes, %	13.0	18.0
Monocytes, %	7.0	4.0
Eosinophils, %	3.0	7.0
Hemoglobin, g/dL	14.7	19.2
Hematocrit, %	41.6	54.3
Platelets, ×10,000/μL	18.8	30.2
Total bilirubin, mg/dL	6.1	3.0
Direct bilirubin, mg/dL	0.1	0.2
Blood urea nitrogen, mg/dL	7.7	8.3
Serum creatinine, mg/dL	0.64	0.50
Aspartate aminotransferase, U/L	37	49
Alanine aminotransferase, U/L	10	23
Lactate dehydrogenase, U/L	595	560
Alkaline phosphatase, U/L	315	493
C-reactive protein, mg/dL	0.35	<0.01
Rapid plasma reagin titer	1:1	Negative
Chest radiograph	Unremarkable	Unremarkable
Bone radiograph	ND	Normal
Hepatomegaly	No	No

*ND, not done.

Case-patient 2 was a 31-year-old woman who came to the same hospital because of a fever in July 2015. One month earlier, she went to another hospital for investigation of a genital ulcer. The day before coming to our hospital, she was given a diagnosis of syphilis and prescribed amoxicillin (1.5 g/d).

A few hours after she took the first dose of amoxicillin, a fever developed, and the patient came to the emergency department of our hospital, where she was given a diagnosis of a Jarisch–Herxheimer reaction. She was also found to be 6 weeks pregnant. Her RPR titer was 1:32 and TPHA titer was 1:160, and she was given a diagnosis of primary syphilis. Three days later, she again came to our hospital for additional evaluation. Treatment was changed to amoxicillin (3 g/d) and probenecid (750 mg/d). Three days after this change in treatment, she could no longer tolerate the medication because of hyperemesis gravidarum, and she was admitted to our hospital. She was given ceftriaxone because she could not tolerate frequent administration of penicillin. Intravenous ceftriaxone (2 g/d) was given for 8 days. Her RPR titer decreased to 1:4 and 1:4 at 6 and 7 months after treatment, respectively. RPR was the last test performed before delivery.

In March 2016, she gave birth to a girl at 39 weeks gestation. The baby did not have any signs or symptoms of congenital syphilis, her RPR titer was negative, and the TPHA titer was 1:320 in a serum sample (Table). The baby was not treated for congenital syphilis, and RPR and TPHA results at 4 months of age showed negative results.

## Conclusions

In 2012, the World Health Organization estimated that 930,000 cases of maternal syphilis resulted in cases of 350,000 congenital syphilis (*1*). In Japan, the National Institute of Infectious Diseases reported that the number of patients with syphilis is increasing (*5*). As the incidence of women with syphilis increases in Japan, incidence of congenital syphilis also increases (*6*). The efficacy of penicillin for treatment of syphilis is well established by clinical experience and is the only treatment option with documented efficacy (*3*).

Both case-patients described in this report were given amoxicillin and probenecid. A pharmacokinetic study reported that oral amoxicillin and probenecid could attain treponemicidal concentrations in cerebrospinal fluid; therefore, these drugs were considered alternative agents for treatment of neurosyphilis (*7*). Tanizaki et al. (*8*) reported that treatment with oral amoxicillin (3 g) and probenecid (750 mg) was highly effective in and well tolerated by syphilis patients with HIV infection. However, in their report, all patients were men.

For case-patient 2, we changed treatment to ceftriaxone, which is active against *T. pallidum* and has an effective concentration in cerebrospinal fluid. Marra et al. (*9*) reported that ceftriaxone is an alternative to penicillin for treatment of neurosyphilis or early syphilis among HIV-infected patients. US Centers for Disease Control and Prevention guidelines recommend ceftriaxone as an alternative treatment of syphilis in nonpregnant women (*3*). However, data regarding the use of ceftriaxone for treatment of maternal infections and prevention of congenital syphilis are insufficient (*3*).

Because RPR titers for both case-patients became nonreactive, treatment with amoxicillin plus probenecid and ceftriaxone successfully prevented syphilis in both fetuses. Amoxicillin and probenecid are not routinely prescribed for pregnant women because of little evidence of their efficacy in preventing congenital syphilis. Because benzathine penicillin G is not available in Japan, intravenous penicillin G is used to treat maternal syphilis. However, this treatment option requires hospitalization for frequent administration; admission of all maternal syphilis patients is not feasible.

Although ceftriaxone can be administered once a day, it requires daily hospital visits. Azithromycin is not recommended for use during pregnancy (*3*), and treatment failures for fetuses have been reported (*10*). Tetracyclines are contraindicated during pregnancy (*3*). Therefore, we used amoxicillin in accordance with guidelines for Japan (*4*).

One study reported the effect of probenecid during pregnancy on fetal outcomes (*11*). Because probenecid can cross the placental barrier, its use in pregnancy must follow careful consideration of anticipated benefits and possible hazards (*12*). Probenecid was prescribed to increase serum levels of penicillin. Amoxicillin monotherapy might be considered for treatment maternal syphilis if an appropriate dose is given.

The World Health Organization estimates that 5.6 million doses of 2.4 million units of benzathine penicillin are needed annually to treat all syphilis cases, and 930,000 doses are needed to prevent all cases of congenital syphilis (*13*). In May 2016, the 69th World Health Assembly reported that benzathine penicillin had been in short supply for several years (*14*). Therefore, during shortages of penicillin, it is prudent to consider alternative treatment regimens.

In conclusion, amoxicillin and ceftriaxone should be considered as alternatives to penicillin for treatment of maternal syphilis. Further studies evaluating the efficacy of amoxicillin and ceftriaxone are warranted.
